# Successful management of a massive subretinal hemorrhage with intravitreal t-PA, perfluoropropane, and bevacizumab: a case report

**DOI:** 10.11604/pamj.2025.51.90.47426

**Published:** 2025-08-11

**Authors:** Nihal El Arari, Saad Benchekroun, Salma Hamidi, Rim El Hachimi, Lalla Ouafae Cherkaou

**Affiliations:** 1 Department of Ophthalmology A, Mohammed V University, Specialty Hospital, Rabat, Morocco

**Keywords:** Subretinal hemorrhage, diabetic retinopathy, perfluoropropane, bevacizumab, case report

## Abstract

Massive subretinal hemorrhage is a rare but vision-threatening complication of proliferative diabetic retinopathy. Rapid and effective intervention is essential to preserve visual function. We report the case of a 40-year-old woman with type 2 diabetes who presented with a sudden, severe bilateral visual acuity drop. Fundoscopy revealed a massive subretinal hemorrhage in the left eye. She was treated with intravitreal injections of tissue plasminogen activator (t-PA), perfluoropropane (C3F8), and bevacizumab. Post-treatment, the hemorrhage resolved with significant visual improvement. Minimally invasive treatment with t-PA, gas tamponade, and anti-VEGF agents is effective in managing massive subretinal hemorrhage and improving visual outcomes.

## Introduction

Diabetic microangiopathy is one of the most common conditions encountered in ophthalmology consultations. It can remain stable if diabetes is well controlled; however, certain factors can exacerbate the condition and lead to severe retinal lesions, such as retinal neovascularization, subretinal or intravitreal hemorrhages, neovascular glaucoma, and retinal detachment. In the case of macular subretinal hemorrhage, the visual prognosis primarily depends on the speed of treatment, the size of the hematoma, and its location. Several treatments can be proposed, ranging from minimally invasive procedures like intravitreal injection of t-PA and perfluoropropane (C3F8) to more invasive endo-ocular surgery with vitrectomy.

## Patient and observation

**Patient information:** a 40-year-old woman presented two days after a sudden drop in visual acuity in her left eye (light perception) and right eye (counting fingers). Medical history includes 10 years of type 2 diabetes treated with insulin and metformin, complicated by proliferative diabetic retinopathy treated with laser, and hypertension treated with amlodipine and losartan.

**Figure 1:**
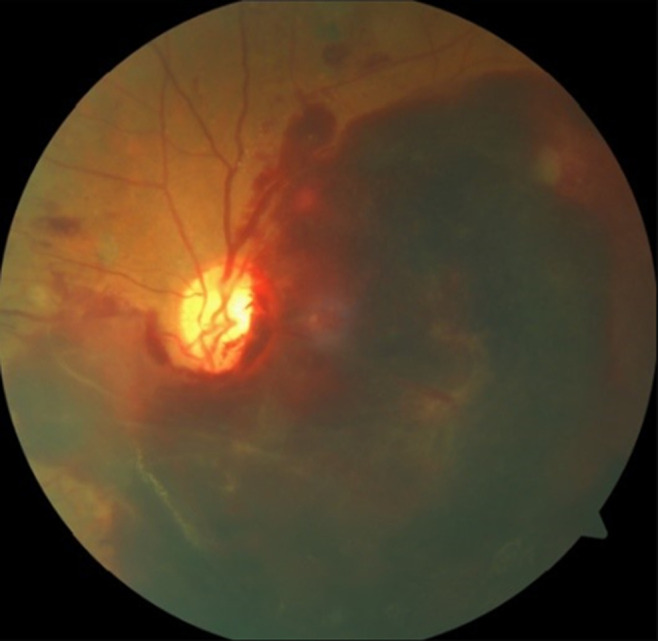
large subretinal hemorrhage in the left eye

**Clinical findings:** on presentation, the patient´s vital signs were within normal limits: blood pressure 130/80 mmHg, heart rate 78 bpm, and temperature 36.7°C. Cardiopulmonary and neurological examinations were unremarkable. Ophthalmic examination revealed a massive hematoma in the left eye obscuring the macula (Figure 1). The right eye showed signs of severe non-proliferative diabetic retinopathy with exudates and hemorrhages.

**Figure 2:**
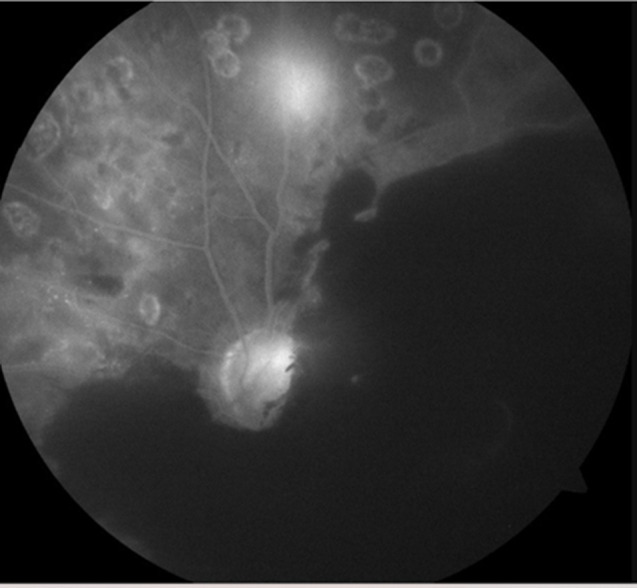
masking effect of hemorrhage with neovascularization and laser marks

**Figure 3:**
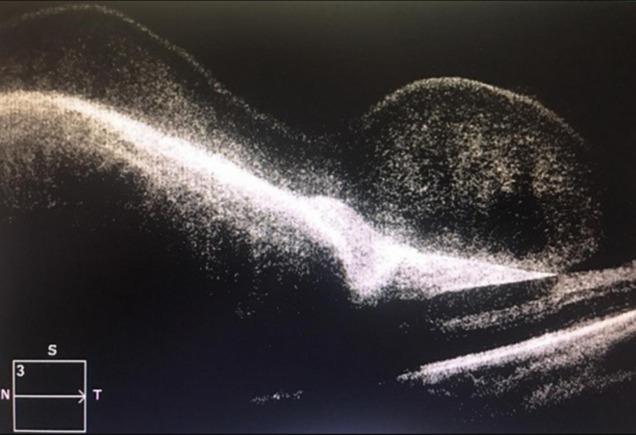
optical coherence tomography showing subretinal hemorrhage in the left eye

**Figure 4:**
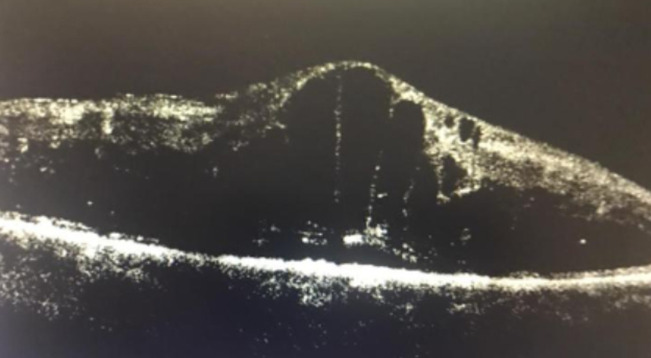
cystoid macular edema in the right eye

**Diagnostic assessment:** fluorescein angiography showed masking by hematoma and neovascularization (Figure 2). Optical coherence tomography confirmed subretinal hemorrhage in the left eye (Figure 3) and cystoid macular edema in the right eye (Figure 4).

**Therapeutic intervention:** intravitreal injections of t-PA, C3F8, and bevacizumab, along with anterior chamber paracentesis.

**Figure 5:**
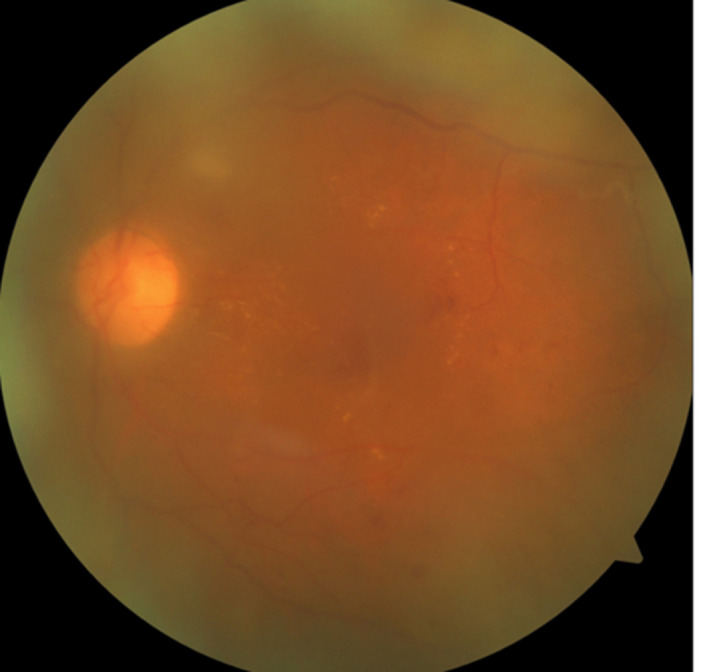
post-treatment fundus showing resolution of hemorrhage

**Figure 6:**
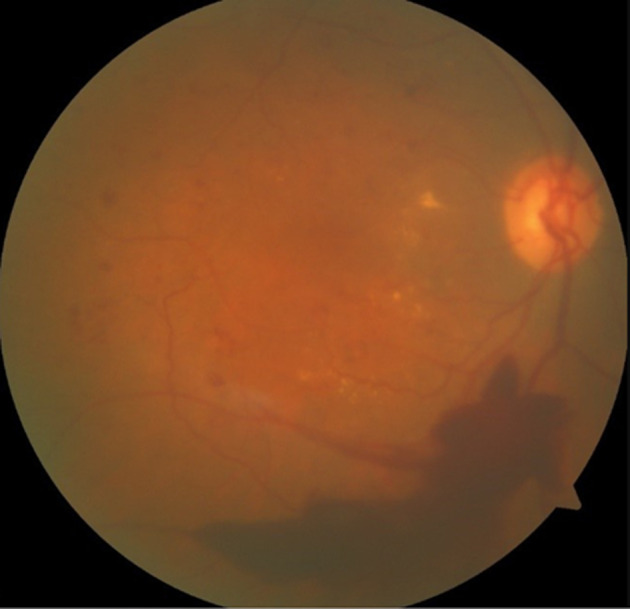
new inferior intraretinal hemorrhage and exudates in the right eye

**Follow-up and outcome:** fundus was obscured on day 1; ultrasound confirmed vitreous hemorrhage. At three months, hemorrhage resolved (Figure 5) with improved visual acuity (20/80). The right eye developed a new hemorrhage (Figure 6) and was treated with pan-retinal photocoagulation and bevacizumab.

**Patient´s perspective:** the patient expressed relief and satisfaction following the treatment, particularly noting the significant improvement in visual acuity. She shared that the sudden loss of vision was distressing, but she felt reassured after receiving prompt and effective care. The patient emphasized the importance of timely medical intervention for conditions such as diabetic retinopathy and subretinal hemorrhage. She is optimistic about the long-term benefits of the treatment.

**Informed consent:** it was obtained from the patient for the publication of this case report and accompanying images. The patient provided written consent, acknowledging that her medical information, photographs, and other related materials would be published for educational and scientific purposes.

## Discussion

Retinal hemorrhages, including those involving the macula, represent a critical turning point in diabetic retinopathy, and the prognosis depends on several factors. To guide therapeutic management and predict functional visual outcomes, the FLATCAPS classification, proposed by Bopp and Mirshahi [1], has been standardized for macular hemorrhages. It incorporates key factors such as foveal involvement, hemorrhage layer, duration, thickness, cause/pathogenesis, and size, offering a thorough approach to assessment and therapeutic decision-making. Recent studies have brought new insights into the management of diabetic macular hemorrhages, especially regarding minimally invasive techniques such as intravitreal injections of tissue plasminogen activator (t-PA) and gas tamponades. These therapies, which we used in this case, continue to demonstrate significant effectiveness in resolving massive subretinal hemorrhages.

**Efficacy of t-PA and gas injections:** intravitreal t-PA has been widely used to treat subretinal hemorrhages, as it promotes fibrinolysis and facilitates the mobilization of the hemorrhage. A study by Novelli *et al*. [2] demonstrated that intravitreal t-PA, when combined with a gas tamponade such as perfluoropropane (C3F8), leads to faster resolution of subretinal hemorrhages and better visual outcomes compared to other treatments. The combination of t-PA with gas agents effectively promotes blood clot displacement and minimizes the need for more invasive procedures, such as vitrectomy. Notably, this approach has shown a lower risk of postoperative complications such as cataracts and retinal detachment, making it a preferable option in many cases of diabetic retinopathy with massive hemorrhages. Moreover, a study by Hassan *et al*. [3] emphasized the benefits of combining t-PA with gas tamponades to improve the structural outcomes in patients with diabetic macular hemorrhages. Their findings support the idea that less invasive procedures like intravitreal injections can provide adequate management and prevent the need for endo-ocular surgery, particularly in the early stages of hemorrhage.

**Combination with anti-VEGF therapy:** another important advancement in managing diabetic macular hemorrhages is the combination of intravitreal t-PA with anti-VEGF therapies such as bevacizumab and ranibizumab. These anti-VEGF agents are effective at reducing retinal neovascularization, which is often a contributing factor to hemorrhage formation in diabetic retinopathy. A study by Nguyen *et al*. [4] found that combining t-PA with anti-VEGF therapy significantly reduces the risk of recurrence of hemorrhages, with additional benefits in terms of reducing neovascular proliferation and enhancing overall visual outcomes. This combination therapy seems particularly effective in treating massive hemorrhages, where neovascularization plays a critical role in disease progression.

**Minimally invasive alternatives:** recent advancements in laser therapy and vitrectomy techniques also contribute to improved management of macular hemorrhages. The study by Huang *et al*. [5] investigated the use of Q-Switched Nd: YAG laser membranotomy for treating macular pre-retinal hemorrhages. Their case series included patients with various etiologies, including diabetic retinopathy. Results showed complete resolution of hemorrhages and significant improvement in visual acuity without serious complications. The authors highlighted the importance of pre-procedure imaging with optical coherence tomography (OCT) to precisely localize the hemorrhage, assess its thickness, and guide safe laser application.

In a complementary study, Karagiannis *et al*. [6] explored the specific role of Nd: YAG laser membranotomy for pre-retinal hemorrhages in patients with diabetic retinopathy. Their findings confirmed the efficacy of this minimally invasive technique in rapidly clearing hemorrhages and improving vision, thereby potentially avoiding more invasive surgical interventions. Additionally, they stressed that pan-retinal photocoagulation (PRP) should be considered before or alongside membranotomy in diabetic patients to address underlying ischemia and reduce the risk of further hemorrhages. In cases where vitrectomy remains necessary, improvements in surgical techniques, such as minimal vitrectomy combined with ILM (internal limiting membrane) peeling and gas tamponades, have reduced the risks of complications like retinal detachment and cataract formation. A study by Sadeghi *et al*. [7] demonstrated that modern vitrectomy techniques, including the use of gas tamponade and anti-VEGF injections, contribute to improved structural and functional outcomes in the management of submacular hemorrhage.

**Long-term management and prevention:** in addition to the acute management of macular hemorrhages, long-term strategies play a crucial role in preventing recurrence. Studies have demonstrated that tight glycemic control, along with regular anti-VEGF therapy, can prevent the progression of diabetic retinopathy and reduce the incidence of subretinal hemorrhages.

## Conclusion

Recent advancements in the treatment of macular hemorrhages in diabetic retinopathy, particularly the combination of t-PA, gas tamponades, and anti-VEGF therapy, have revolutionized the management of these complex cases. As demonstrated in our case, intravitreal injections of t-PA and perfluoropropane provided an excellent outcome, with complete resolution of the hemorrhage and significant improvement in visual acuity. These minimally invasive approaches should be considered as first-line treatments, especially in patients with massive hemorrhages, before progressing to more invasive options like vitrectomy, which carries a higher risk of complications. As newer research continues to refine these therapies, the prognosis for patients with diabetic macular hemorrhages continues to improve.
